# Effects and safety of auricular acupressure on depression and anxiety in isolated COVID-19 patients: A single-blind randomized controlled trial

**DOI:** 10.3389/fpsyt.2022.1041829

**Published:** 2022-12-05

**Authors:** Wa Cai, Kun Zhang, Guan-Tao Wang, Jin Li, Xiang-Yu Wei, Wen Ma, Ya-Juan Li, Bo Wang, Wei-Dong Shen

**Affiliations:** ^1^Department of Acupuncture, Shanghai Shuguang Hospital Affiliated to Shanghai University of Traditional Chinese Medicine, Shanghai, China; ^2^Institute of Acupuncture and Anesthesia, Shanghai Shuguang Hospital Affiliated to Shanghai University of Traditional Chinese Medicine, Shanghai, China; ^3^Department of Traditional Chinese Medicine, Lianyang Community Health Service Center, Shanghai, China

**Keywords:** auricular acupressure, depression, anxiety, COVID-19, randomized controlled trial

## Abstract

**Objective:**

Psychological distress such as depression and anxiety resulted from coronavirus disease 2019 (COVID-19) have attracted increasing attention. The aim of this randomized controlled trial is to evaluate the effects and safety of auricular acupressure on depression and anxiety in isolated COVID-19 patients.

**Methods:**

68 participants diagnosed with COVID-19 pneumonia (18–80 years old, SDS ≥ 50, SAS ≥ 45) were recruited and randomly allocated to the auricular acupressure group and the sham auricular acupressure group by a computer-generated random number sequence from 9th June to 30th June 2022. The group allocation was only blinded to the participants. Those in the auricular acupressure group were attached magnetic beads against 4 auricular points Shenmen, Subcortex, Liver and Endocrine, while sham group used four irrelevant auricular points. Outcomes were measured by Zung Self-Rating Depression Scale (SDS) and Zung Self-Rating Depression Scale (SAS) before and after treatment in both groups through electronic questionnaire in mobile phones.

**Results:**

After treatment, statistically significant differences were found in scores of SAS in both groups (*P* < 0.001 in auricular acupressure group; *P* = 0.003 in sham group), and SDS scores reduced significantly in the auricular acupressure group (*P* = 0.002). Significant reduced SAS and SDS scores were achieved in the auricular acupressure group than that in the sham group (F = 4.008, *P* = 0.049, MD −7.70 95% CI: −9.00, −6.40, SMD −2.79 95% CI: −3.47, −2.11 in SDS; F = 10.186, *P* = 0.002, MD −14.00 95% CI: −15.47, −12.53, SMD −4.46 95% CI: −5.37, −3.56 in SAS). No adverse events were found in either group during the whole study.

**Conclusion:**

Auricular acupressure is an effective and safe treatment for alleviating symptoms of depressive and anxiety in patients with COVID-19.

**Clinical trial registration:**

https://www.chictr.org.cn//, identifier ChiCTR2200061351.

## Introduction

COVID-19 is an infectious disease caused by the severe acute respiratory syndrome coronavirus 2 (SARS-CoV-2). The first known case of COVID-19 was identified in Wuhan, China, in December 2019. With worldwide spread, the disease led to the COVID-19 pandemic. Reported by World Health Organization (WHO), the number of confirmed cases of COVID-19 has increased to over 521 million including more than 6,274 thousand deaths as of 20 May 2022 (1).

Globally, the psychological effects of coronavirus disease (COVID-19) have drawn more attention (2, 3). Isolation with direct contact only to medical and nursing staff, COVID-19 patients experienced physical discomfort, loneliness and psychosocial stressors, which can cause depression, anxiety and other psychological problems (2, 4). Patients infected with COVID-19 may experience psychological distress due to existential factors such as physical discomfort, uncertainty about returning to work, loss of income. Reported by previous studies, isolation or living alone was associated with higher risk of anxiety or depression (5, 6). Ongoing lock-down in some regions have delayed hospitalization schedules, such as routine chemotherapy of cancer patients or routine hemodialysis of patients with renal failure. Other key factors including the massive media coverage of the global outbreak and the risk of new variants of SARS-CoV-2 also lead to the increase of psychological disturbances. Researchers found that 80–90% of COVID-19 patients had loss of smell and taste (7–9), which increased the risk of anxiety and depression (10, 11). As a result, high rates of depression and anxiety have been reported in patients with COVID-19 (12–16). A recent study (17) evaluated COVID-19 survivors and found a percentage of 31% in depression and 42% in anxiety. Another study used by online survey found that 34% of COVID-19 patients had symptoms of anxiety, depression and an increased risk of suicide (18).

Antidepressants and antianxietics are the widely acknowledged treatment of depression and anxiety respectively. Whereas, these medications brought along obvious side effects, such as headache, blurred vision and gastrointestinal reactions (19, 20).

As a result, other therapeutic strategies with minor side effects should be explored to alleviate depression and anxiety of isolated COVID-19 patients. Auricular acupressure is a Chinese traditional complementary and alternative therapy by placing a tiny magnetic bead on the selected acupressure point of the ears to stimulate the meridians. Previous studies (21–26) have revealed that auricular acupressure treatment can effectively alleviate depression or anxiety. However, no prospective studies were announced so far to evaluate the treatment of auricular acupressure targeting psychological disturbances caused by COVID-19. As a result, this study was conducted to investigate the safety and efficacy of auricular acupressure on depression and anxiety in isolated patients with COVID-19.

## Methods

### Study design

**Figure 1:**
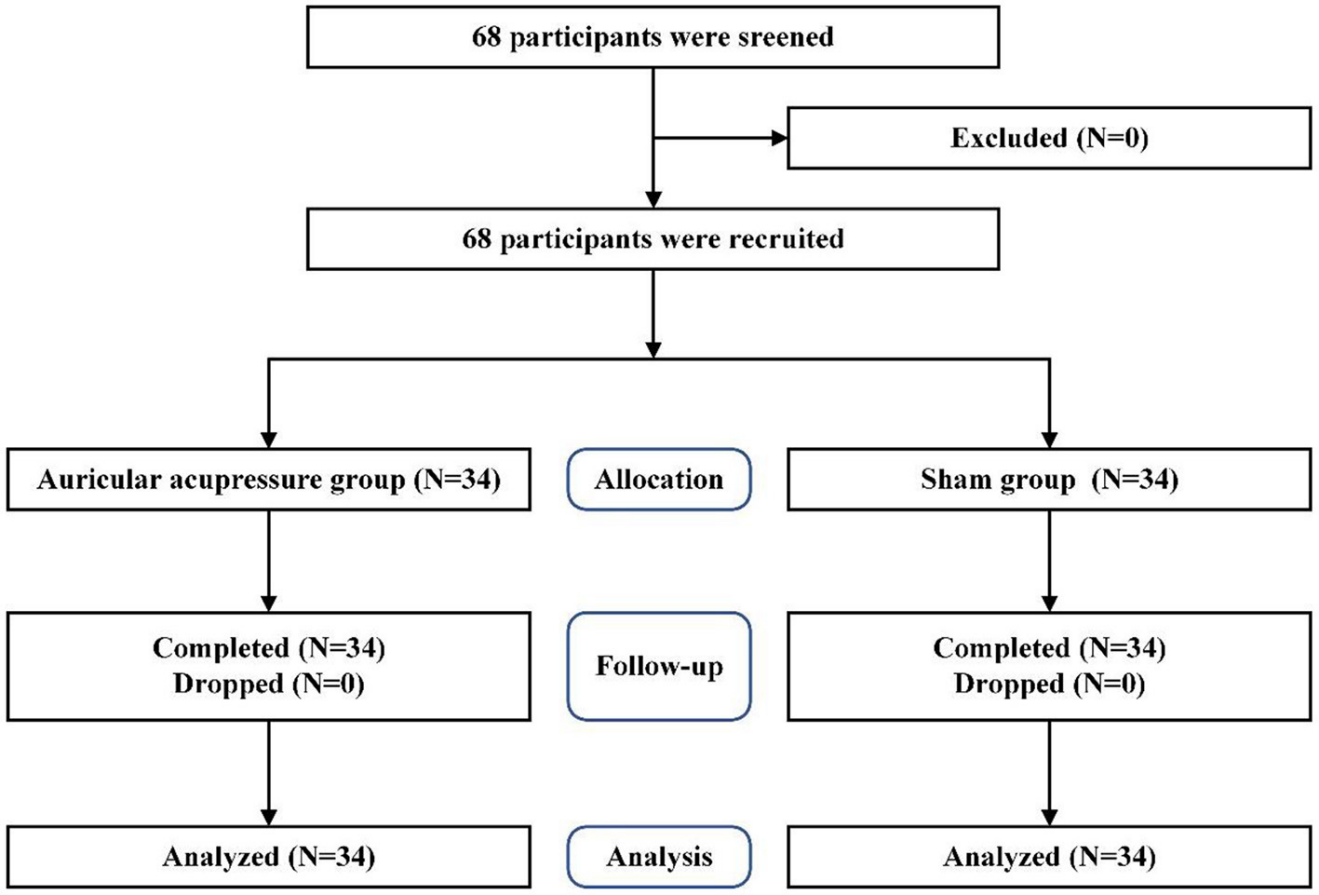
CONSORT flow diagram of the study.

A randomized, sham-controlled, single-blind study design was performed in this study according to the CONSORT guidelines (27). The flow chart of the trial was presented in Figure 1.

### Ethics statement

The study was conducted in accord with the principles of the Declaration of Helsinki. The trial was approved by Ethics Committee of Shuguang Hospital affiliated with Shanghai University of Traditional Chinese Medicine on 5th May 2022 (certificate No.2022-1116-53-01). The study was registered in Chinese Clinical Trial Registry (No. ChiCTR2200061351).

### Participants

Participants were recruited in Shanghai Huangpu COVID-19 Modular Hospital and Shanghai Shuguang Hospital from 9th June to 30th June 2022. All the participants volunteered for the study and met with the inclusion criteria as follows: (1) diagnosis of COVID-19 pneumonia based on clinical manifestations, abnormal findings in computed tomography scans and confirmation by real-time reverse transcriptase polymerase chain reaction assays from nasal and pharyngeal swab specimens; (2)aged 18–80 years old; (3) an index score of 50 or higher on the Zung Self-Rating Depression Scale (SDS); (4) an index score of 45 or higher on the Zung Self-Rating Anxiety Scale (SAS); (5) able to read and fill out the scales. The exclusion criteria included: (1) self-reported history of severe mental illness; (2) not taking any antidepressants or anxiolytics within the 1 month before admission; (3) serious dysfunction of heart, lung, liver or kidney; (4) severe cognitive dysfunction; (5) dysphasia or aphasia.

### Sample size, blinding and randomization

The G-Power 3.1 was used to calculate the sample size of this study. A previous study (28) presented that the effect size was 0.78, power (1- β) was 0.80 and the significance level (α) was 0.05. The required number of participants in each group was 30. In the study, 34 participants were placed in each group considering a dropout rate of 10%.

The study applied a single-blind method to the recruited participants to ensure that they did not know their group allocation. An independent researcher was in charge of the procedure of randomization. A permuted block randomization was used to ensure an equal number in each group. Allocations were sealed in opaque envelopes. Another researcher opened the envelopes to allocate participants to two different groups.

### Interventions

Participants in both groups received routine medical care including psychological support, medication administration and diet control.

### Auricular acupressure group

**Figure 2:**
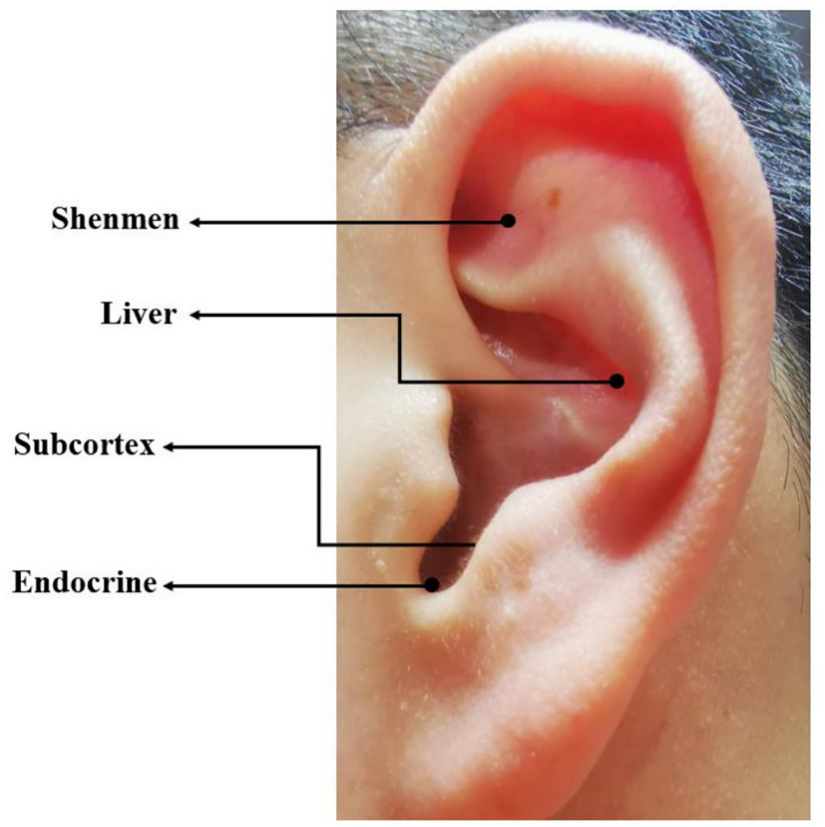
Auricular points selected in the auricular acupressure group.

Certified auricular therapists disinfected the external ear of participants with 75% ethanol, then attached a piece of adhesive plaster (7 mm × 7 mm) with a tiny magnetic bead (2 mm × 2 mm) by a pincette to the selected auricular points of both ears. According to the Chinese Standard Ear Acupoints Chart Nomenclature and Location of Auricular Points (GB/T-13734–2008), four auricular points Shenmen, Subcortex, Liver and Endocrine were located by the 13-cm round-headed spring auricular probe (Figure 2). These auricular points were selected due to their effectiveness in relieving depression and anxiety according to the classical theory of TCM (29). Participants were required to press the magnetic beads against the 4 auricular points 1 min 5 times a day for 14 days. The ear magnetic beads were changed every 3 days.

### Sham group

**Figure 3:**
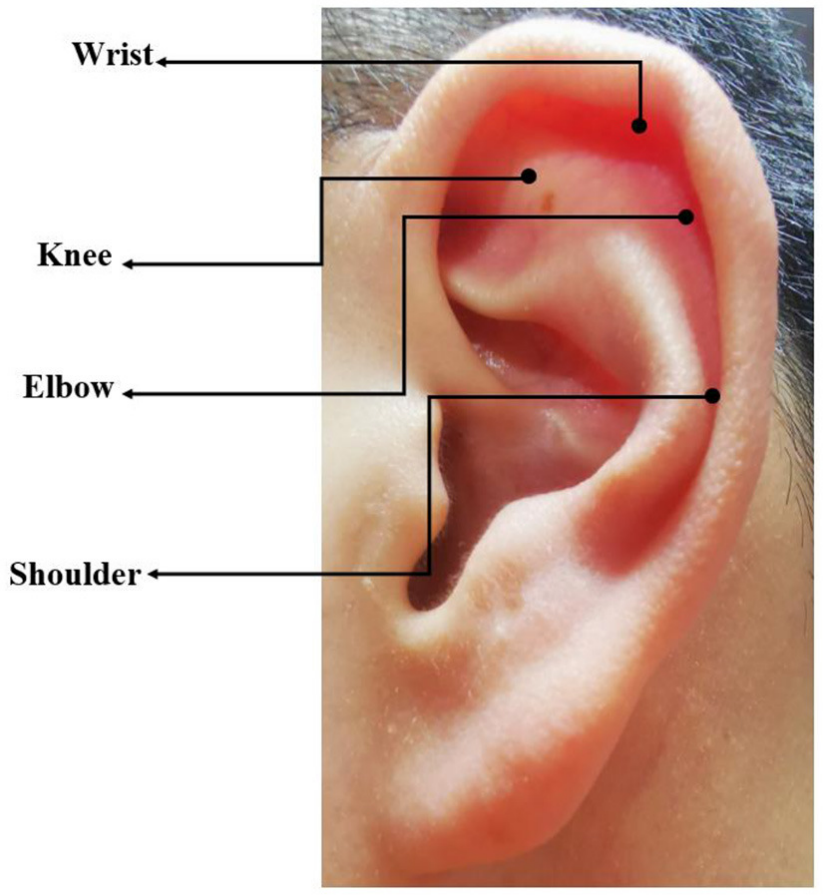
Auricular points selected in the sham group.

All operations and procedures were the same as the auricular acupressure group except the selection of auricular acupoints. The following auricular points were used in sham group: Knee, Wrist, Elbow, Shoulder (Figure 3) which had no therapeutic effect for depression and anxiety according to literature review and clinical experience.

### Outcomes

The study applied Self-Rating Depression Scale (SDS) to assess depression severity, while Self-Rating Anxiety Scale (SAS) was used to evaluate level of anxiety through electronic questionnaire in mobile phones.

### SDS

The SDS was a 20-item self-assessment scale based on the emotional, psychological and physical symptoms related to depression (30). The scale could monitor changes in depression severity over time in the study. Each item was scored from 1 to 4 on a 4-level scale. SDS index scores are classified as normal (< 50), mild depression (50 to 59), moderate to marked major depression (60 to 69), and severe to extreme major depression (>70) (30). The Chinese version of SDS was reported to have a Cronbach's alpha of 0.86 and the test-retest reliability (3-week) was 0.83 (31).

### SAS

The SAS was a 20-item self-report scale designed to measure anxiety levels according to 4 groups of manifestations including cognitive, autonomic, motor and central nervous system symptoms (32). Each question was scored on a Likert-type scale of 1–4. The index score range of 20–44, 45–59, 60–74 and ≥75 indicated normal, mild to moderate anxiety, severe anxiety, and extreme anxiety respectively (33). A previous study revealed that the Chinese version of SAS had strong internal consistency (a = 0.92) with a Cronbach's alpha of 0.78 and a split-half reliability of 0.75 (34).

### Safety assessment

Adverse events (AEs) were assessed at each visit during the whole study. Related AEs (skin infection, damage, or allergic reaction of the ear) would be reported and provided with appropriate treatment. Participants could withdraw from the study at any time for any reason.

### Data analysis

Statistical analyses were conducted using SPSS version 24.0 (SPSS, Inc., Chicago, IL, USA). Continuous variables were presented as mean ± SD, while categorical variables were presented as a number (%). Confirming the normality of the data, the one-way analysis of variance (ANOVA) was applied for data between groups. The paired *T*-test was used for the pre- and post- data within each group. Mean difference (MD) and standardized mean difference (SMD) were used to measure the effect size for the intervention compared to sham control to find its' clinical significance. The study calculated 95% confidence intervals (CIs). *P*-values < 0.05 were considered statistically significant.

## Results

### Participants' general characteristics

Sixty eight participants were screened, recruited and randomly allocated to two groups. No one dropped out or withdrew in this study. All the recruited participants completed the study. There found no significant differences in demographic characteristics between different groups (*p* > 0.05) (Table 1).

**Table 1:** Participants' demographic characteristics.

**Baseline characteristics**	**Auricular acupressure group (*n* = 34)**	**Sham group (*n* = 34)**	***P*-value**
Gender, *n* (%)			0.808^Δ^
Male	15 (44.1)	16 (47.1)	
Female	19 (55.9)	18 (52.9)	
Age: Mean (years) (SD)	62.1 (16.8)	58.8 (13.3)	0.364^▴^
Marital status, *n* (%)			0.813^Δ^
Single	2 (5.9)	3 (8.8)	
Married	29 (85.3)	27 (79.4)	
Divorced or widowed	3 (8.8)	4 (11.8)	

n, number of subjects; SD, standard deviation.

Δ Chi-squared test; ^▴^ANOVA.

### Effects of auricular acupressure

#### Depression

Table 2 presented the within- and between-group comparisons of SDS scores. No significant differences were found in the SDS scores between the two groups before treatment (F = 0.179, *P* = 0.674). The SDS scores decreased in both groups after 14 days of treatment with a significant change in the auricular acupressure (AA) group (56.6 ± 2.7 vs. 45.7 ± 2.0, *P* = 0.002 in AA group; 54.9 ± 2.9 vs. 53.4 ± 3.3, *P* = 0.248 in sham group). Compared with the sham group, the auricular acupressure group had greater improvement in change scores of SDS (F = 4.008, *P* = 0.049; MD −7.70, 95% CI: −9.00, −6.40; SMD −2.79, 95% CI: −3.47, −2.11).

**Table 2:** Results of SDS in auricular acupressure and sham groups.

	**Auricular acupressure group**	**Sham group**				
	Mean ± SD	Mean ± SD	ANOVA F	ANOVA P	MD (95% CI)	SMD (95% CI)
Pre-treatment	56.6 ± 2.7	54.9 ± 2.9	0.179	0.674	/	/
Post-treatment	45.7 ± 2.0	53.4 ± 3.3	4.008	0.049^*^	−7.70 (-9.00,−6.40)	−2.79 (-3.47,−2.11)
Paired *T*-test P	0.002^**^	0.248				

ANOVA, analysis of variance; MD, mean difference; SD, standard deviation; SMD, standardized mean difference.

Data are presented as mean ± SD. The data between two groups were measured by one-way ANOVA. The pre- and post- data within each group were assessed by paired T-test.

* p < 0.05,

** p < 0.01.

### Anxiety

The change of SAS scores was shown in Table 3. Also, the SAS scores between the two groups before treatment had no significant differences (F = 0.076, *P* = 0.784). Both groups had significant decrease in the SAS scores after treatment (56.3 ± 4.0 vs. 34.4 ± 1.8, *P* < 0.001 in AA group; 54.6 ± 4.3 vs. 48.4 ± 4.0, *P* = 0.003 in sham group). The change of SAS scores in auricular acupressure group was more significant than that in the sham group (F = 10.186, *P* = 0.002; MD −14.00, 95% CI: −15.47, −12.53; SMD, −4.46, 95% CI: −5.37, −3.56).

**Table 3:** Results of SAS in auricular acupressure and sham groups.

	**Auricular acupressure group**	**Sham group**				
	Mean ± SD	Mean ± SD	ANOVA F	ANOVA P	MD (95% CI)	SMD (95% CI)
Pre-treatment	56.3 ± 4.0	54.6 ± 4.3	0.076	0.784	/	/
Post-treatment	34.4 ± 1.8	48.4 ± 4.0	10.186	0.002^**^	−14.00 (-15.47,−12.53)	−4.46 (-5.37,−3.56)
Paired *T*-test P	< 0.001^**^	0.003^**^				

ANOVA, analysis of variance; MD, mean difference; SD, standard deviation; SMD, standardized mean difference.

Data are presented as mean ± SD. The data between two groups were measured by one-way ANOVA. The pre- and post- data within each group were assessed by paired T-test.

** p < 0.01.

### Adverse events

No adverse events were found in either group during the whole period of study, which indicated that auricular acupressure is a safe treatment for depression and anxiety in isolated COVID-19 patients.

## Discussion

Our study showed that auricular acupressure on appropriate points could significantly alleviated anxiety and depression of isolated COVID-19 patients. Numerous studies have revealed that auricular acupressure treatment is effective with emotional disorders (23, 24, 26, 35, 36). It was reported in a study (37) that transcutaneous vagus nerve stimulation of auricular point can significantly modulate the resting state functional connectivity (rsFC) of default mode network (DMN) which is closely related with emotion. Another study (24) demonstrated that auricular acupressure treatment could significantly decreased cortisol level and increased serotonin levels. Secreted from the HPA (hypothalamic-pituitary-adrenal) axis, cortisol is a physiological biomarker of stress level. It has been confirmed in most studies (38–41) that higher levels of serotonin activity lead to decreased depression and anxiety.

The study also revealed that sham group could reduce the symptoms of depression and anxiety after 14 days of intervention, which might be explained by that all the participants received routine medical care including psychological support, the methods of which mainly focused on having conversation with patients by encouraging them, publicizing the basic knowledge, rehabilitation plan and prognosis of COVID-19 to reduce their worry and concern. Psychotherapy was found to improve patients' depression, insomnia and quality of life (42). Furthermore, the recovery of COVID-19 also contributed to the alleviation of depression and anxiety.

Auricular acupressure is one of the most frequently-used traditional Chinese therapies due to its advantages of no trauma and convenient use (43). The therapy intends to place a tiny magnetic bead over the appropriate point on the ears. The therapeutic effect is determined by the selection of auricular points. Four auricular points were selected as listed: Shenmen, Subcortex, Liver and Endocrine were particularly effective to relieve depression and anxiety based on the classical theory and clinical experience of TCM, while the sham group used auricular points of Knee, Wrist, Elbow, Shoulder which have no relations with the treatment of mental disorders. All the physical or psychological disturbances are caused by an imbalance of body's energy or *qi* based on TCM theory. As the basic life energy, *qi* flows through the whole body. Auricular acupressure can regulate *qi* and the functions of the visceras and treat various dysfunction or diseases by stimulating the auricular points.

The study was the first prospective, randomized, sham-controlled and single-blind trial announced so far to assess the efficacy of auricular acupressure treatment for depression and anxiety caused by COVID-19. However, several limitations of the study still need to be mentioned as follows: First, considering the COVID-19 pandemic, the study was limited in sample size. Second, a double-blind approach was not achieved in the study because the researcher knew the auricular points. Third, a researcher-administered outcome scale was lacked in the study limited by patient-reported outcome measurement.

## Conclusion

In conclusion, the study found that auricular acupressure treatment is significantly effective for alleviating symptoms of depressive and anxiety in patients with COVID-19 indicated by the SDS and SAS scores. The therapy is safe and easily applicable for COVID-19 patients. Further studies are needed to clarify the mechanisms of the anti-depressive, anti-anxiety effects of auricular acupressure treatment.

## Data availability statement

The original contributions presented in the study are included in the article/supplementary material, further inquiries can be directed to the corresponding authors.

## Ethics statement

The studies involving human participants were reviewed and approved by the Ethics Committee of Shuguang Hospital Affiliated with Shanghai University of Traditional Chinese Medicine. The patients/participants provided their written informed consent to participate in this study.

## Author contributions

The study was designed by WC and W-DS. KZ, G-TW, JL, X-YW, WM, Y-JL, and BW performed the study. WC analyzed the data and drafted the manuscript. All authors read and approved the final manuscript.

## Funding

The study was funded by the Training Plan of Prestigious Traditional Chinese Medicine Doctors in Pudong New Area (Grant No. PWRzm2020), the research project of omicron contracted COVID-19 from Shanghai University of Traditional Chinese Medicine (Grant No. 2022YG-55, 2022YG-56, and 2022YG-57). National Natural Science Foundation of China (Grant No. 82004444), Shanghai Municipal Health Commission (Grant No. 20204Y0472), the Youth Medical Talents-Specialist Program of Shanghai Rising Stars of Medical Talents Youth Development Program, Shanghai Municipal Health Commission (Grant No. 2021LPTD-004), Shanghai Municipal Health Commission (Grant No. ZY (2021–2023)-0209–10), Clinical technology innovation project of municipal hospital (Grant No. SHDC22021210), Shanghai Science and Technology Commission (Grant No. 20Y21902900).

## Conflict of interest

The authors declare that the research was conducted in the absence of any commercial or financial relationships that could be construed as a potential conflict of interest.

## Publisher's note

All claims expressed in this article are solely those of the authors and do not necessarily represent those of their affiliated organizations, or those of the publisher, the editors and the reviewers. Any product that may be evaluated in this article, or claim that may be made by its manufacturer, is not guaranteed or endorsed by the publisher.
